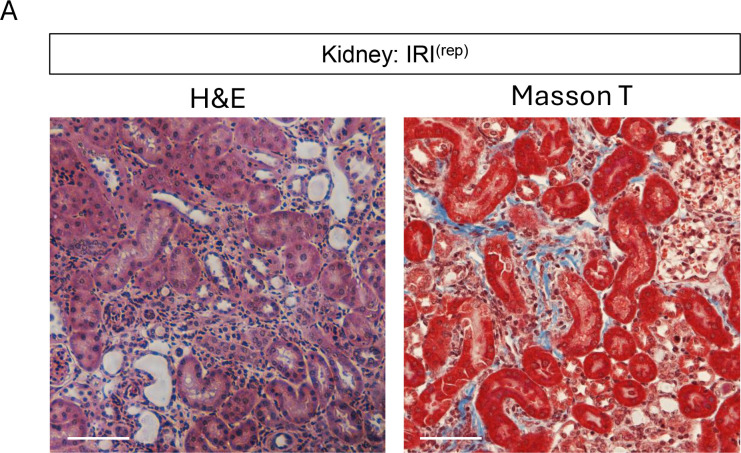# Repetitive ischemic injuries to the kidneys result in lymph node fibrosis and impaired healing

**DOI:** 10.1172/jci.insight.198650

**Published:** 2025-09-23

**Authors:** Omar H. Maarouf, Mayuko Uehara, Vivek Kasinath, Zhabiz Solhjou, Naima Banouni, Baharak Bahmani, Liwei Jiang, Osman A. Yilmam, Indira Guleria, Scott B. Lovitch, Jane L. Grogan, Paolo Fiorina, Peter T. Sage, Jonathan S. Bromberg, Martina M. McGrath, Reza Abdi

Original citation: *JCI Insight*. 2018;3(13):e120546. https://doi.org/10.1172/jci.insight.120546

Citation for this corrigendum: *JCI Insight*. 2025;10(18):e198650. https://doi.org/10.1172/jci.insight.198650

The authors recently became aware that that the MECA79-stained KLN:WT panel of Figure 3D is incorrect and was inadvertently uploaded during figure assembly. In addition, the Masson T-stained Kidney: IRI(rep) in supplemental Figure 2A was an inadvertently duplicated from the representative Kidney: LTbr-Ig panel in Figure 4A. The correct figure panels are shown below. The HTML, PDF, and Supplemental Data have been updated online. The authors regret the error.

## Figures and Tables

**Figure 3D.**
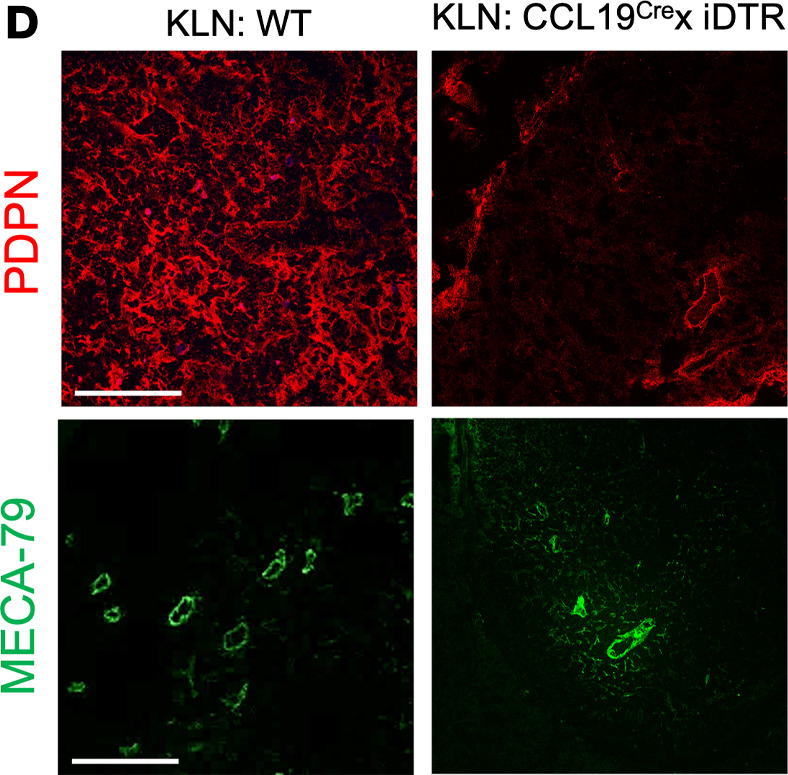


**Supplemental Figure 2A.**